# In Vitro Propagation of *Eleutherine palmifolia* (L.) Merr.: Optimization of Surface Sterilization and Effects of BA and NAA on Shoot and Root Induction

**DOI:** 10.1155/tswj/2774274

**Published:** 2025-10-06

**Authors:** Jatuporn Hongthongkham, Charinrat Srivarom, Naphat Joomdok, Surachai Rattanasuk

**Affiliations:** Department of Science and Technology, Faculty of Liberal Arts and Science, Roi Et Rajabhat University, Selaphum, Roi Et, Thailand

**Keywords:** Clorox concentration, *Eleutherine palmifolia*, explant survival, in vitro propagation, root induction, shoot induction, surface sterilization

## Abstract

*Eleutherine palmifolia* (L.) Merr., or Wan Hom Daeng, is a medicinal plant valued for its antioxidant, anti-inflammatory, and antibacterial properties. Its bulbs contain flavonoids, phenolics, and naphthoquinones, but slow natural propagation and low seed viability hinder large-scale cultivation and conservation. This study evaluated the optimal Clorox concentration and exposure time for sterilizing *E. palmifolia* bulb explants and examined the effects of BA and NAA on in vitro shoot and root development. The results revealed that surface sterilization with 20% Clorox for 20 min yielded the highest explant survival rate at 73.33%. Shoot induction was significantly enhanced in MS medium supplemented with 2 or 4 mg/L BA, or a combination of 2 mg/L BA and 0.5 mg/L NAA, all achieving a 100% shoot formation rate. Root induction was most effective in MS medium containing 0.5 mg/L NAA alone, also with a 100% success rate. These findings establish a reliable and efficient protocol for the in vitro propagation of *E. palmifolia*, providing a foundation for its conservation, sustainable use, and potential commercial cultivation to meet increasing demand in the medicinal plant sector.

## 1. Introduction

*Eleutherine palmifolia* (L.) Merr., commonly known as Wan Hom Daeng in Thailand, is a medicinal plant native to Southeast Asia. It is traditionally used by the Dayak community in Kalimantan, Indonesia, and is also valued in Thai folk medicine [1]. Traditionally, its bulbs have been employed to treat various ailments, including diabetes, breast cancer, nasal congestion, and fertility issues [2, 3]. Phytochemical investigations have revealed that *E. palmifolia* contains essential compounds such as flavonoids, which contribute to its therapeutic properties. Notably, studies have demonstrated the plant's potential to induce apoptosis and inhibit the cell cycle in breast cancer cells, underscoring its promise as an anticancer agent [4].

Despite its recognized medicinal value, the conventional propagation of *E. palmifolia* remains challenging due to limited seed viability and inherently slow growth rates. To overcome these constraints, plant tissue culture techniques, particularly micropropagation, present effective alternatives for the rapid and large-scale production of this species [5]. Micropropagation is a critical tool for the efficient propagation of economically and pharmacologically important plants, offering several advantages such as year-round cultivation, genetic uniformity, and space-efficient production under controlled environmental conditions, independent of external climatic factors. However, the commercial application of micropropagation is not without challenges. Key obstacles include technical complexities, biological and physiological limitations, genetic instability, and operational concerns such as insufficient infrastructure, a shortage of trained personnel, and the potential risks associated with overproduction. Addressing these issues is imperative to realize the full potential of micropropagation in advancing commercial horticulture and agricultural biotechnology [6].

The application of micropropagation to *E. palmifolia* necessitates the optimization of culture conditions, including the selection of appropriate explants, sterilization protocols, and the composition of growth media supplemented with specific plant growth regulators (PGRs). Such optimization is crucial for enhancing shoot induction, callus formation, and rooting efficiency [7]. Recent advancements in plant tissue culture, such as the integration of nanoparticles, have further improved micropropagation efficiency by reducing contamination and enhancing callus induction [8]. Therefore, developing an efficient in vitro propagation protocol for *E. palmifolia* is essential to meet the growing demand for this medicinal plant and to support its conservation and sustainable utilization.

A few studies on the micropropagation of *Eleutherine* species have been published. Hoesen [9] reported the successful vegetative propagation of *Eleutherine* sp. (Iridaceae) using bulb explants cultured on Murashige and Skoog (MS) medium supplemented with various PGRs. Callus formation was effectively induced using picloram at 1 mg/L in combination with 2,4-dichlorophenoxyacetic acid (2,4-D) at concentrations of 0.5 and 1 mg/L. The highest number of shoot buds was observed in media containing 2 mg/L benzyladenine (BA) without the addition of naphthalene acetic acid (NAA). Optimal root formation was achieved in MS medium supplemented with both BA and NAA. Notably, MS medium containing 1 mg/L picloram and 1 mg/L 2,4-D yielded the highest frequency of callus induction (100%) and produced the largest callus diameter. During the acclimatization stage, all regenerated plantlets (100%) survived and were successfully transplanted into soil under field conditions. Based on the information above, the objectives of this study were (1) to determine the optimal concentration and exposure duration of Clorox for effective surface sterilization of *Eleutherine* sp. explants and (2) to evaluate the effects of different concentrations of the PGRs BA and NAA on shoot and root induction under in vitro conditions.

## 2. Materials and Methods

### 2.1. Determining the Optimal Concentration of Clorox and Duration for Surface Sterilization

*E. palmifolia* rhizomes were collected from Ban Thamuang, Selaphum, Roi Et, Thailand. The rhizomes were washed twice with tap water to remove the soil. The outer leaf sheaths were peeled off (1–2 layers). The rhizomes were then washed with dishwashing liquid by shaking for 5 min, followed by rinsing with clean water. Surface sterilization began by immersing the rhizomes in 70% ethanol for 2 min. They were then treated with Clorox solutions at concentrations of 0%, 10%, 15%, 20%, and 25% for 15 and 20 min, respectively. Following disinfection, the explants were rinsed three times with sterile distilled water, each rinse lasting 5 min. The disinfected plant materials were transferred to sterile petri dishes and further trimmed by peeling off 3–5 additional layers of leaf sheath. Each explant was vertically divided into one smaller section and cultured on standard MS medium. The cultures were maintained at 25°*C* ± 2°C with a light intensity of 3000 lux and a photoperiod of 16 h/day. The survival percentage of the explants was recorded after 60 days of culture.

### 2.2. Effects of PGRs BA and NAA on Shoot Induction

Sterilized rhizome explants of *E. palmifolia* were vertically sliced into individual sections. Each explant was cultured on modified MS medium supplemented with various concentrations of BA: 0, 1, 2, and 4 mg/L, both alone and in combination with 0.5 mg/L of NAA (as shown in Table 1). The cultures were incubated at 25°*C* ± 2°C under 3000 lux light intensity with a 16-h photoperiod. Each treatment was conducted in four replicates, with eight explants per replicate. After 60 days of culture, the percentage of shoot formation, the number of shoots per explant, and the average shoot length were recorded.

### 2.3. Effects of PGRs BA and NAA on Root Induction

Sterilized rhizome explants were vertically divided into single segments and cultured on modified MS medium supplemented with BA at concentrations of 0, 1, 2, and 4 mg/L, both alone and in combination with 0.5 mg/L of NAA (see Table 1). The cultures were maintained at 25°*C* ± 2°C with a light intensity of 3000 lux and a 16-h photoperiod. The experiment was replicated four times with eight explants per MS medium formula. After 60 days of culture, data were collected on the percentage of root induction, the number of roots per shoot, and root length.

### 2.4. Data Analysis

This research employed a completely randomized design (CRD). A one-way analysis of variance (ANOVA) was conducted to compare the data, followed by Duncan's multiple range test (DMRT) for post hoc analysis. Statistical significance was set at *p* < 0.05.

## 3. Results and Discussion

### 3.1. Effect of Clorox Concentration and Duration for Surface Sterilization

Rhizomes of *E. palmifolia* were surface sterilized using Clorox at concentrations ranging from 0% to 25% for durations of 15 and 20 min, followed by culture on MS medium for 60 days. The highest explant survival rate (73.33%) was observed with 20% Clorox treatment for 20 min. In contrast, increasing the Clorox concentration to 25% for either 15 or 20 min resulted in a reduced survival rate compared to the 20% treatments. Specifically, explants treated with 20% Clorox for 15 and 20 min exhibited survival rates of 40% and 73.33%, respectively (Table 2). The findings of this study demonstrate the importance of optimizing surface sterilization and culture media composition for the successful micropropagation of *E. palmifolia*. Among the tested sterilization treatments, 20% Clorox for 20 min provided the highest explant survival rate (73.33%), indicating an effective balance between microbial decontamination and tissue viability. Higher concentrations (25%) led to increased tissue damage and reduced explant viability [10, 11], supporting previous reports that excessive exposure to strong sterilants can be phytotoxic [12].

### 3.2. Effect of BA and NAA on Shoot Induction

**Figure 1:**
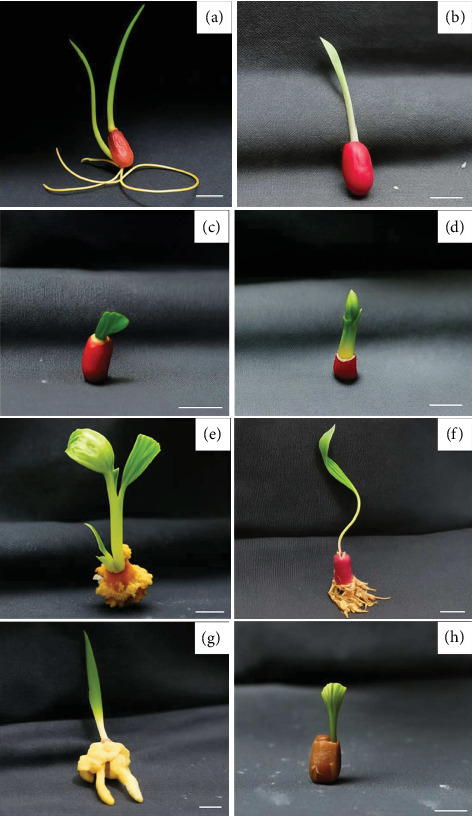
Shoot formation characteristics of *E. palmifolia.* (a) MS medium without plant growth regulators. (b) MS medium supplemented with 1 mg/L BA. (c) MS medium supplemented with 2 mg/L BA. (d) MS medium supplemented with 4 mg/L BA. (e) MS medium supplemented with 0.5 mg/L NAA. (f) MS medium supplemented with 1 mg/L BA and 0.5 mg/L NAA. (g) MS medium supplemented with 2 mg/L BA and 0.5 mg/L NAA. (h) MS medium supplemented with 4 mg/L BA and 0.5 mg/L NAA (scale bars 1 cm).

Sterilized rhizome explants of *E. palmifolia* were cultured on modified MS medium supplemented with various concentrations of BA (0–4 mg/L), as well as combinations of BA (0–4 mg/L) and NAA (0.5 mg/L), for 60 days to induce shoot formation. All media formulations supported shoot induction, though with varying efficiencies. Notably, MS media supplemented with 2 and 4 mg/L BA, and 2 mg/L BA combined with 0.5 mg/L NAA, resulted in the highest shoot induction rate of 100% (Table 3). Regarding shoot proliferation, the highest average number of shoots per explant (1.5 shoots/explant) was observed in explants cultured on MS medium without PGRs. This treatment also yielded the greatest average shoot length, measuring 4.67 cm (Figure 1). Shoot induction was successfully achieved in all treatments containing BA and BA+NAA, with the best results (100% shoot induction) occurring in media with 2 or 4 mg/L BA alone and 2 mg/L BA combined with 0.5 mg/L NAA. These results align with prior studies that emphasized the role of BA in enhancing shoot initiation by promoting cytokinin activity [13–16]. Interestingly, the highest shoot proliferation and shoot length were observed in explants cultured without any PGRs, suggesting that *E. palmifolia* rhizomes possess intrinsic hormonal cues that support initial shoot development under suitable basal medium conditions [17].

**Figure 2:**
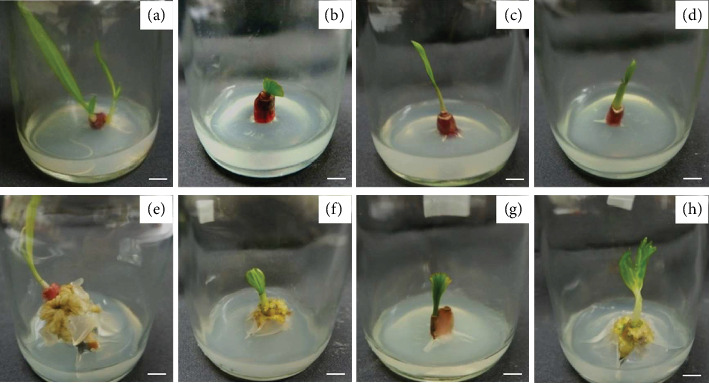
Callus formation characteristics of *E. palmifolia*. (a) MS medium without plant growth regulators. (b) MS medium supplemented with 1 mg/L BA. (c) MS medium supplemented with 2 mg/L BA. (d) MS medium supplemented with 4 mg/L BA. (e) MS medium supplemented with 0.5 mg/L NAA. (f) MS medium supplemented with 1 mg/L BA and 0.5 mg/L NAA. (g) MS medium supplemented with 2 mg/L BA and 0.5 mg/L NAA. (h) MS medium supplemented with 4 mg/L BA and 0.5 mg/L NAA (scale bars 0.5 cm).

In addition, callus formation was observed in *E. palmifolia* rhizomes, particularly when cultured on MS medium supplemented with NAA alone or in combination with 0.5 mg/L NAA and either 1 or 4 mg/L BA (Table 4). Among the treatments, MS medium supplemented with 1 mg/L BA and 0.5 mg/L NAA induced the highest callus formation rate at 70%. Meanwhile, MS medium containing only 0.5 mg/L NAA resulted in the highest average fresh and dry weights of callus at 0.58 and 0.33 g, respectively (Figure 2). Callus formation was significantly influenced by auxin-rich media [18].

MS medium supplemented with 0.5 mg/L NAA alone yielded the highest fresh and dry weights of callus, while the combination of 1 mg/L BA and 0.5 mg/L NAA induced the highest callus formation rate (70%). These observations are consistent with studies highlighting the synergistic effects of auxin and cytokinin combinations on callus induction [19]. The accumulation of biomass in the NAA-only treatment may be attributed to its role in stimulating cell enlargement and division in undifferentiated tissues [20, 21].

### 3.3. Effect of BA and NAA on Root Induction

**Figure 3:**
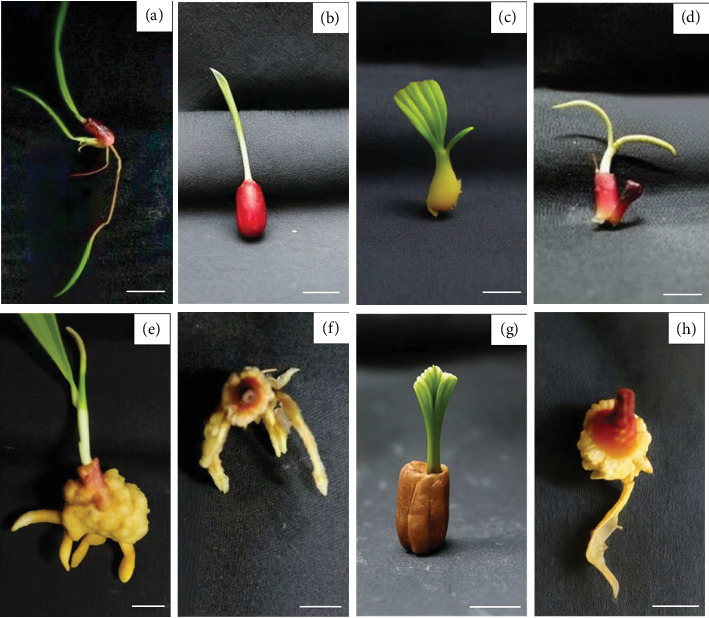
Root formation characteristics of *E. palmifolia*. (a) MS medium without plant growth regulators. (b) MS medium supplemented with 1 mg/L BA. (c) MS medium supplemented with 2 mg/L BA. (d) MS medium supplemented with 4 mg/L BA. (e) MS medium supplemented with 0.5 mg/L NAA. (f) MS medium supplemented with 1 mg/L BA and 0.5 mg/L NAA. (g) MS medium supplemented with 2 mg/L BA and 0.5 mg/L NAA (scale bars 1 cm). (h) MS medium supplemented with 4 mg/L BA and 0.5 mg/L NAA.

Shoots of *E. palmifolia* derived from rhizome explants cultured on MS medium without PGRs for 60 days were subsequently transferred to MS medium supplemented with various concentrations of BA (0–4 mg/L), as well as combinations of BA (0–4 mg/L) with 0.5 mg/L NAA, and cultured for an additional 60 days to induce rooting. The most effective medium for root induction was MS supplemented with 0.5 mg/L NAA, which resulted in the highest root induction percentage (100%) and the greatest average number of roots per shoot at 3.75 ± 1.58 roots/shoot (Table 5 and Figure 3).

In the rooting phase, MS medium with 0.5 mg/L NAA was the most effective, yielding 100% root induction and the highest average number of roots per shoot. This confirms the efficacy of low-concentration auxin treatments for root initiation, as also reported in micropropagation protocols of other medicinal plants [22, 23]. Collectively, the study establishes a reliable protocol for the in vitro propagation of *E. palmifolia* using rhizome explants. The findings are valuable for the conservation and mass propagation of this medicinally important plant, particularly where wild populations are threatened or propagation by conventional means is limited.

## 4. Conclusions

This study demonstrated an effective protocol for the in vitro propagation of *E. palmifolia* through tissue culture techniques. Surface sterilization using 20% Clorox for 20 min yielded the highest explant survival rate at 73.33%. Shoot induction was most successful on MS medium supplemented with 2–4 mg/L BA alone or 2 mg/L BA combined with 0.5 mg/L NAA, achieving a 100% shoot formation rate. Although the highest number and length of shoots were observed in hormone-free MS medium, callus formation was most effectively induced using 1 mg/L BA with 0.5 mg/L NAA, while the highest callus biomass was obtained with 0.5 mg/L NAA alone. Rooting was optimally induced on MS medium containing only 0.5 mg/L NAA, producing a 100% rooting rate with an average of 3.75 roots and 4.67 cm root length per shoot. These findings provide a reliable micropropagation system for *E. palmifolia* and can be applied to support the conservation and commercial cultivation of this medicinally valuable species.

## Figures and Tables

**Table 1:** Modified MS medium supplemented with various concentrations of plant growth regulators BA and NAA.

**MS medium formula**	**Plant growth regulators**	
	**BA (mg/L)**	**NAA (mg/L)**
1	0	0
2	1	0
3	2	0
4	4	0
5	0	0.5
6	1	0.5
7	2	0.5
8	4	0.5

**Table 2:** Effect of Clorox concentration and exposure duration on the survival percentage of *E. palmifolia* rhizome explants.

**Duration of sterilize (minutes)**	**Clorox concentration (%)**	**S** **u** **r** **v** **i** **v** **a** **l** **percentage** (%) ± **SD**
15	0	0.00 ± 0.00^b^
	10	13.33 ± 9.08^bc^
	15	26.67 ± 11.81^bc^
	20	40.00 ± 13.10^b^
	25	33.33 ± 12.60^bc^

20	0	0.00 ± 0.00^b^
	10	20.00 ± 10.69^bc^
	15	40.00 ± 13.09^b^
	20	73.33 ± 11.82^a^
	25	20.00 ± 10.70^bc^

*Note:* Means followed by different letters within a column are significantly different at the 95% confidence level (*p* < 0.05).

**Table 3:** Percentage of shoot induction, number of shoots per explant, and shoot length of *E. palmifolia* cultured on MS medium supplemented with various concentrations of BA and NAA for 60 days.

**Plant growth regulators (mg/L)**		**S** **h** **o** **o** **t** **formation** (%) ± **SD**	**A** **v** **e** **r** **a** **g** **e** **number** of **shoots** (**shoots**/**explant**) ± **SD**	**A** **v** **e** **r** **a** **g** **e** **shoot** **length** (**cm**) ± **SD**
**BA**	**NAA**			
0	0	75.00 ± 46.29^ab^	1.50 ± 1.19^a^	4.67 ± 3.11^a^
1	0	87.50 ± 35.35^ab^	1.00 ± 1.06^ab^	2.37 ± 2.37^ab^
2	0	100.00 ± 0.00^a^	1.00 ± 0.00^ab^	1.00 ± 0.65^b^
4	0	100.00 ± 0.00^a^	1.00 ± 0.00^ab^	1.75 ± 0.26^b^
0	0.5	50.00 ± 53.45^b^	0.58 ± 0.52^b^	2.80 ± 4.47^ab^
1	0.5	75.00 ± 46.29^ab^	1.25 ± 1.16^ab^	2.40 ± 2.02^ab^
2	0.5	100.00 ± 0.00^a^	1.00 ± 0.00^ab^	2.12 ± 1.32^ab^
4	0.5	50.00 ± 53.45^b^	0.51 ± 0.52^b^	0.77 ± 1.14^b^

*Note:* Means followed by different letters within a column are significantly different at the 95% confidence level (*p* < 0.05).

**Table 4:** Percentage of callus formation, fresh weight, and dry weight of *E. palmifolia* cultured on MS medium supplemented with various concentrations of BA and NAA for 60 days.

**Plant growth regulators (mg/L)**		**C** **a** **l** **l** **u** **s** **formation** (%) ± **SD**	**A** **v** **e** **r** **a** **g** **e** **fresh** **weight** (**g**) ± **SD**	**A** **v** **e** **r** **a** **g** **e** **dry** **weight** (**g**) ± **SD**
**BA**	**NAA**			
0	0	0.00 ± 0.00^b^	0.00 ± 0.00^b^	0.00 ± 0.00^b^
1	0	0.00 ± 0.00^b^	0.00 ± 0.00^b^	0.00 ± 0.00^b^
2	0	0.00 ± 0.00^b^	0.00 ± 0.00^b^	0.00 ± 0.00^b^
4	0	0.00 ± 0.00^b^	0.00 ± 0.00^b^	0.00 ± 0.00^b^
0	0.5	50.00 ± 53.45^a^	0.58 ± 0.82^a^	0.33 ± 0.50^a^
1	0.5	70.00 ± 46.29^a^	0.32 ± 0.29^ab^	0.11 ± 0.11^b^
2	0.5	0.00 ± 0.00^b^	0.00 ± 0.00^b^	0.00 ± 0.00^b^
4	0.5	12.50 ± 35.35^b^	0.12 ± 0.12^b^	0.01 ± 0.03^b^

*Note:* Means followed by different letters within a column are significantly different at the 95% confidence level (*p* < 0.05).

**Table 5:** Rooting percentage, number of roots per shoot, and root length of *E. palmifolia* cultured on MS medium supplemented with various concentrations of BA and NAA for 60 days.

**Plant growth regulators (mg/L)**		**R** **o** **o** **t** **i** **n** **g** **percentage** (%) ± **SD**	**A** **v** **e** **r** **a** **g** **e** **number** of **roots** (**roots**/**shoot**) ± **SD**	**A** **v** **e** **r** **a** **g** **e** **root** **length** (**cm**) ± **SD**
**BA**	**NAA**			
0	0	75.00 ± 46.29^ab^	3.25 ± 2.05^a^	3.28 ± 2.15^a^
1	0	0.00 ± 0.00^d^	0.00 ± 0.00^b^	0.00 ± 0.00^c^
2	0	0.00 ± 0.00^d^	0.00 ± 0.00^b^	0.00 ± 0.00^c^
4	0	0.00 ± 0.00^d^	0.00 ± 0.00^b^	0.00 ± 0.00^c^
0	0.5	100.00 ± 0.00^a^	3.75 ± 1.58^a^	1.90 ± 0.30^b^
1	0.5	25.00 ± 46.29^cd^	0.25 ± 0.46^b^	0.17 ± 0.32^c^
2	0.5	0.00 ± 0.00^d^	0.00 ± 0.00^b^	0.00 ± 0.00^c^
4	0.5	50.00 ± 53.45^bc^	0.75 ± 1.38^b^	0.71 ± 0.76^c^

*Note:* Means followed by different letters within a column are significantly different at the 95% confidence level (*p* < 0.05).

## Data Availability

The datasets generated during and/or analyzed during the current study are available from the corresponding author upon reasonable request.
